# Phonetic-DeepKANet: a robust audio spoofing detection framework for English and Arabic

**DOI:** 10.1038/s41598-026-51950-9

**Published:** 2026-05-09

**Authors:** Muteb Aljasem, Hafsa Ilyas, Ali Javed, Jasim Alnahas, Mohammed Abouheaf

**Affiliations:** 1 Department of Electronics and Computer Engineering, Robotics Engineering at Bowling Green State University, Bowling Green, OH 43403 USA; 2 Department of Software Engineering, University of Engineering and Technology, Taxila, 47050 Pakistan; 3 Department of Industrial Engineering, Faculty of Engineering, University of Tabuk, 47512 Tabuk, Saudi Arabia; 4 Robotics Engineering at Bowling Green State University, Bowling Green, OH 43403 USA

**Keywords:** Audio spoofing for Arabic and English, ASVspoof, Kolmogorov Arnold network, Multi-view features, Partial spoofing, Phonetic-DeepKANet, Engineering, Mathematics and computing

## Abstract

Audio spoofing attacks, specifically deepfakes, are massively used these days to compromise the security of automatic speaker verification-based systems, leading to data breaches and financial scams. Existing audio spoofing countermeasures are not well-generalized and experience issues when detecting unknown spoofing attacks, including deepfake. Moreover, Arabic audio spoofing detection has been largely neglected, primarily due to the scarcity of Arabic language spoofing datasets. This paper proposes a novel dual-modality approach, Phonetic-DeepKANet (PDK-Net), capable of reliable detection of audio spoofing attacks in English and Arabic. The proposed PDK-Net is comprised of a deep feature extraction module incorporating TransRawNet (TR-Net), an acoustic–phonetic feature extraction module, and a Kolmogorov Arnold Network (KAN) classifier. The deep features from TR-Net are complemented with multi-view acoustic–phonetic representations through concatenation and then classified using KAN. This paper also introduces an Arabic audio spoofing dataset to address the limited availability of such datasets and advance the research in audio spoofing detection for underrepresented languages. The proposed method is evaluated utilizing ASVspoof-2019 LA, 2021 LA, and DF, partial spoof, and our Arabic audio spoofing dataset created in this work. Extensive experimentation on multiple datasets, including voice conversion and text-to-speech synthesized samples, algorithm-wise and cross-corpora evaluation demonstrates the effectiveness and generalizability of our method. We attained the best min-tDCF of 0.09 and 0.14 on the ASVspoof-2019 LA and ASVspoof-2021 LA datasets, respectively, compared to baseline models. However, for the Arabic spoofing dataset, the PDK-Net achieved an EER of 8.06%. It is noteworthy that our method performed best for detecting LA attacks over all 41 methods reported in the ASVspoof-2021 challenge. Further, our method registered the third-best EER of 17.55% amongst 33 challenge participants on the ASVspoof-2021 DF set. These results demonstrate the effectiveness and improved generalization of our approach while detecting unknown spoofing attacks, including codec compressions, channel variations, and encoding artifacts.

## Introduction

An automatic speaker verification (ASV) system serves as a biometric tool to authenticate the speaker identities based on the voice. ASV systems have become an integral part of various application domains, including voice biometrics, over-the-phone banking, electronic payments, and voice assistants. Although the ASVs have provided various advantages and authenticated the identity, such systems are vulnerable to spoofing attacks, including impersonation, synthetic speech, voice conversion, and replay attacks. The advanced and sophisticated spoofing attacks, including deepfake attacks, further challenged the reliability of ASV systems. These spoofing attacks stimulate data and security breaches, unauthorized access to sensitive information, and financial fraud. This significantly raises concerns regarding the security and reliability of voice-based identification.

**Figure 1:**
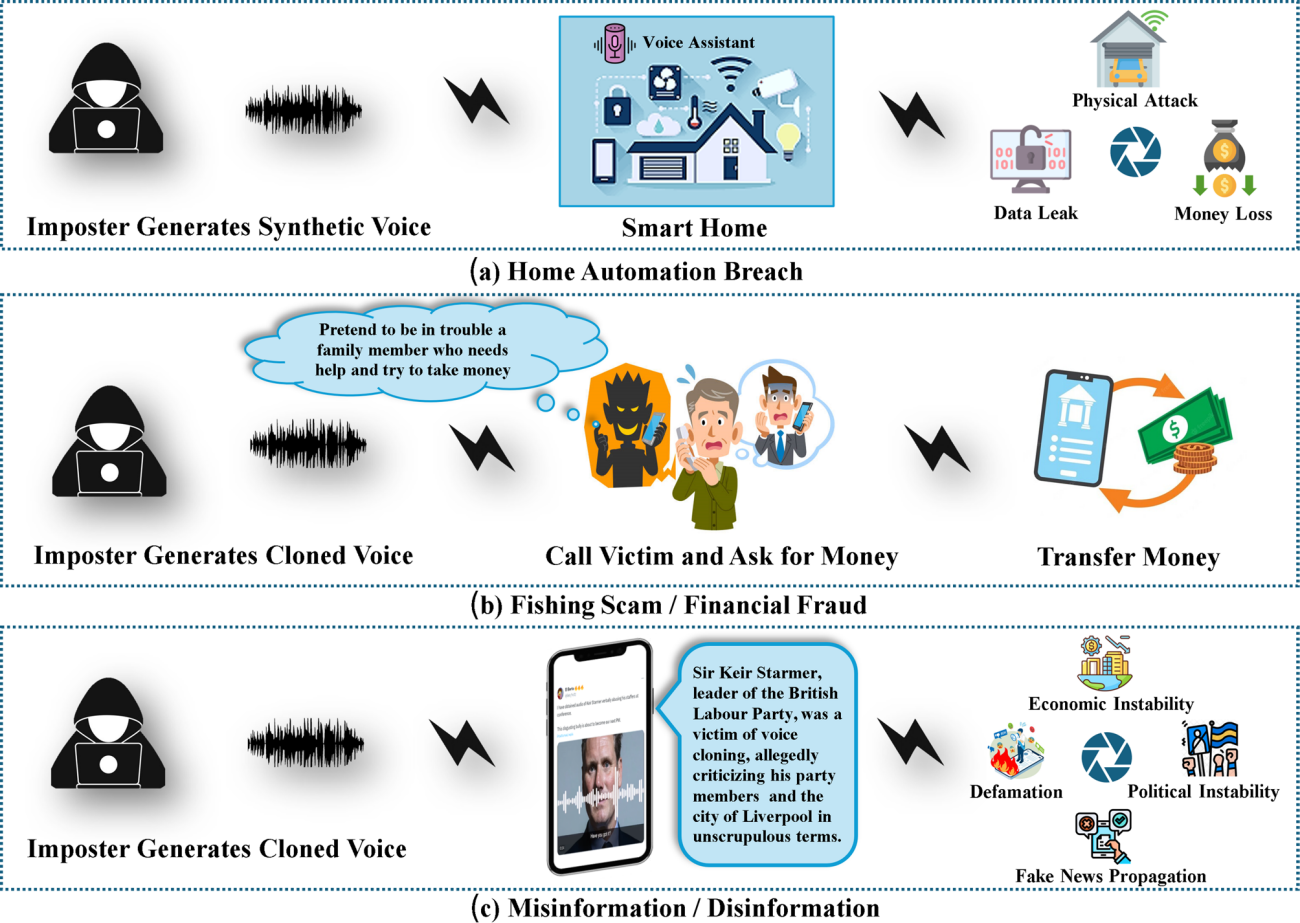
A few scenarios of audio spoofing attacks.

Spoofing attacks are direct attacks that are executed without the target ASV systems’ knowledge of their own architecture. These attacks are also referred to as black box attacks and are considered a great threat to the security of speaker verification systems since direct spoofing attacks are easy to execute. Spoofing attacks can be generally categorized as physical access (PA) and logical access (LA) attacks. Physical access attacks refer to conventional replay attacks where the audio recording of the victim can be used to breach the speaker verification systems. However, logical access attacks involve the use of synthesized voices to attack ASV systems. Such synthesized voices are widely generated through text-to-speech (TTS) and voice conversion (VC) algorithms. Voice conversion approaches utilize the natural voice to create synthetic audio with the human voice attributes and thus are more difficult to identify. Additionally, due to the advanced TTS and VC techniques, realistic and indistinguishable human voices can now be generated, enabling the LA attacks to pose significant security threats to the ASV systems. Along with the spoofing attacks on ASV systems, audio deepfakes have also emerged as a great threat to society these days. The synthesized or impersonated voice of a person can be generated using artificial intelligence (AI) algorithms and then can be used for spreading disinformation, financial scams, security breaches, and unauthorized access. The evolution and availability of AI tools and methods have enabled individuals to generate audio deepfakes effortlessly. For instance, Resemble AI^1^ is a publicly available tool to create audio deepfakes, and the Microsoft VALL-E model can generate the cloned voice with a three-second audio sample^2^. A few cases of audio spoofing attacks are depicted in Fig. 1. For instance, Fig. 1a illustrates the security breaches of smart homes, which can lead to unauthorized access to the home and can cause multiple losses, such as financial loss, privacy violations, physical danger, sensitive data leakage, loss of control over the home environment, psychological harm, etc. Likewise, a scenario of a fishing scam is shown in Fig. 1b, where the convincing cloned voice can be utilized to deceive the person into transferring money. Along with the scams and frauds, the cloned voices of renowned personalities can be used to create and spread virulent hate speech and can lead to political instability, intolerance, and social conflicts (Fig. 1c). Consequently, the detection of such audio deepfakes has now become a crucial need in this modern world. Furthermore, to enhance the robustness of ASV systems against sophisticated logical attacks, it is now essential to develop spoofing countermeasures with the ability to identify impersonated voices and spoofing attacks that compromise speaker verification systems.

Researchers have introduced various spoofing detection frameworks, from the conventional hand-crafted features-based methods to the recent deep neural network-based approaches. The conventional feature-based descriptors^3–6^ have proved to be effective for detecting voice spoofing attacks, mainly utilizing the ASVspoof datasets. However, such conventional approaches are incapable of capturing the complex variations that exist in the diverse spoofed audios generated using unknown algorithms. With the advent of neural networks, various deep learning (DL) methods have been introduced that learn complex speech features and improve the generalizability of spoofing attack detection frameworks to some extent. DL-based methods have applied different techniques for the detection of spoofed audio, such as one-class learning models^7–13^, methods that analyze raw audio samples^14–16^, approaches using audio spectral features^17,18^, and methods that process the 2D-audio spectrograms^19–22^. To determine the centroid vector utilizing only bonafide data, a one-class learning method is demonstrated in^11^, where the centroid was adaptively shifted via computing the weighted average of bonafide representations. StateNet^14^ was a multi-task learning framework based on RawNet2 and the amalgamation of spatio-temporal features. Awais et al.^18^ introduced the spectral-temporal features fusion-based approach, where the amalgamation of spectral and temporal coefficients generating spectral-temporal deviated coefficients was passed to the auto-encoder-decoder-based architecture to identify the spoofing cues. In^23^, AVFakeNet, comprising a transformer-based architecture, was introduced that utilized mel-spectrogram images of audio signals for distinguishing between spoofed and bonafide utterances. Even though the literature has demonstrated various detection methods, the audio spoofing detection domain still has many challenges that need to be addressed. In general, existing DL models are prone to adversarial attacks, where the addition of a small perturbation to the audio sample causes the model to incorrectly classify the audio. Additionally, the performance on the unknown spoofing attacks, along with deepfake, needs to be further improved. Lastly, the performance of spoofing countermeasures in cross-corpora scenarios needs massive improvement.

It has been observed that the existing works mainly focus on the spoofing detection models for the English language; however, only a few existing studies have focused on the detection of synthetic Arabic audio. Zaynab et al.^24^ are the first to explore the detection of fake Arabic audio. This study^24^ introduced a single-speaker Arabic audio dataset and an Arabic-AD method based on self-supervised learning to detect imitated and synthetic audio. A single speaker dataset cannot accurately evaluate the robustness of the spoofing detection model. Sahar et al.^25^ evaluated the neural network-based model on their dataset, which comprised English and mixed (English and Arabic) real and fake audios collected from social media sites.^26^ conducted research focusing on the detection of AI-generated Arabic speech. For this, the model was trained on their own dataset of English audio samples and then evaluated on single-speaker Arabic and English audio samples (their own created dataset). These methods^24–26^ utilize small Arabic datasets that are not publicly available. Additionally, the approaches^24,26^ are evaluated on a single-speaker Arabic dataset, indicating the lack of diversity and generalizability in terms of speaking style and accent. The existing literature highlights the huge research gap regarding Arabic audio spoofing datasets and spoofing detection models.

To overcome the existing challenges, a novel spoofing detection framework, namely Phonetic-DeepKANet (PDK-Net), is introduced that particularly enhances English and Arabic audio spoofing detection. Additionally, we also introduced a novel Arabic spoofing dataset, including the spoofed audios generated using different voice conversion algorithms. PDK-Net is designed to learn the generalized multi-view features from distinct dimensions, including deep features, phonetic duration, and wave2vec dimensions. Particularly, PDK-Net incorporates a deep feature module employing TransRawNet (TR-Net) for the extraction of complex deep features. Additionally, our framework also encompasses an acoustic–phonetic feature extraction module for learning acoustic and phonetic features. The features are concatenated to represent a comprehensive feature vector and then classified through the Kolmogorov Arnold Network (KAN). Along with the enhanced performance for LA and deepfake attacks, PDK-Net also demonstrates satisfactory performance for the partially spoofed utterances, perturbed samples, and spoofed Arabic speech utterances. The main contributions of this work are:

- We proposed a generalized Phonetic-DeepKANet framework, complementing deep features with the multi-view acoustic–phonetic features for the precise detection of known and unknown audio spoofing attacks.
- Incorporated the cutting-edge Kolmogorov Arnold Network that learns adaptive, feature-wise spline-based transformations to improve performance for the unknown spoofing attacks.
- Introduced a novel multi-speaker Arabic audio spoofing dataset incorporating the spoofed audio generated using voice conversion techniques.
- Conducted extensive experiments on multiple datasets, including cross-corpora evaluation, to demonstrate the effectiveness of the proposed PDK-Net while distinguishing the bonafide and spoofed utterances.
- Our method outperforms all 41 participating methods of the ASVspoof-2021 challenge for detecting LA attacks and third-best performer for the detection of deepfake attacks among the 33 participating methods.

The rest of the paper is structured as follows: "Literature review" section presents the literature review, providing a detailed analysis of the existing approaches. The proposed methodology is demonstrated in "Proposed methodology" section, whereas details of datasets, evaluation metrics, and extensive experimentation are demonstrated in "Experimental results" section. In "Discussion" section, the discussion is presented, and lastly, the conclusion is provided in "Conclusion" section.

## Literature review

This section examines the existing approaches presented in the literature for the effective detection of spoofed and bonafide audio samples. The approaches are categorized as conventional approaches, one-class learning methods, multi-view feature fusion-based deep learning framework, 2D spectrogram image-based approaches, and other spoofed audio detection frameworks. An overview of the reviewed methodologies is presented in Table 1; however, a detailed discussion is provided in the subsequent subsections.

**Table 1:** Overview of spoofed audio detection approaches.

Year	Approach	Dataset	Results	Limitations
Conventional Approaches for Spoofed Audio Detection				
2021	GTCC, MFCC, spectral centroid, flux + BiLSTM^3^	ASVspoof-2019 (LA)	EER = 3.05	Computationally complex May fail to generalize well on unseen attacks
2021	ELTP-LFCC + DBiLSTM^4^	ASVspoof-2019 (LA)	EER = 0.74, min-tDCF = 0.008	
2022	ATP-GTCC + SVM^5^	ASVspoof-2019 (LA, PA) VSDC	2019 LA EER = 0.1, min-tDCF = 0.015 2019 PA EER = 1.1, min-tDCF = 0.069 VSDC EER = 0.6, min-tDCF = 0.04	
2024	GTCC spectrogram features + ResNet50^6^	ASVspoof-2019 (LA, PA) VIHL	2019 LA, EER = 0.6 2019 PA, EER = 1.15 VIHL, EER = 4.3	
One-class Learning-based Methods				
2021	one-class softmax loss function^7^	ASVspoof-2019 (LA)	EER = 2.19, min-tDCF = 0.059	The trainable centroid vector representation is influenced by spoofed data
2023	AESVVDD—Autoencoder and one-class support vector data distribution loss^9^	ASVspoof-2019 (LA) In the wild FoR dataset	2019 LA EER = 8.10 In the wild EER = 15.08, min-tDCF = 0.059 FoR dataset EER = 23.34	Variations in bonafide speech due to speaker, environment, and channel differences are neglected
2023	SAMO—Speaker attractor multi-center one-class learning^10^	ASVspoof-2019 (LA)	EER = 1.08 min-tDCF = 0.0356	Performance on the ASVspoof-2021 dataset remains unexplored
2024	One-class learning approach based on adaptive centroid shift^11^	ASVspoof-2019 (LA) ASVspoof-2021(LA, DF)	2019 LA EER = 0.17, min-tDCF = 0.0050 2021 LA EER = 1.30, min-tDCF = 0.2172 2021 DF, EER = 2.19	Performance against adversarial attacks remains untested
2024	Knowledge distillation-based one-class learning method^12^	ASVspoof-2019 (LA) ASVspoof-2021(LA, DF) In the wild	2019 LA, EER = 0.39 2021 LA, EER = 0.90 2021 DF, EER = 2.27 In the wild, EER = 7.68	This method is not solely based on one-class embedding learning
2024	one-class learning and knowledge distillation-based method^13^	ASVspoof-2019 (LA)	2019 LA EER = 1.89, min-tDCF = 0.049	Performance is yet to be explored for ASVspoof-2021 dataset
2025	Spectro-temporal network incorporating KLOC-softmax^27^	ASVspoof-2019 (LA) ASVspoof-2021 (LA, DF)	2019 LA EER = 0.38, min-tDCF = 0.0127 2021 LA EER = 3.61, min-tDCF = 0.2583 2021 DF EER = 16.62	Performance degradation for complex environments Generalizability needs to be enhanced
Multi-view Features Fusion-Based Deep Learning Frameworks				
2023	BTS-E—Breathing Talking Silence Encoder^28^	ASVspoof-2021 (LA)	EER = 9.44, min-tDCF = 37.67	The results need to be further improved
2023	SpoTNet-Spoofing aware Transformer^17^	ASVspoof-2019 (LA)	EER = 0.95, min-tDCF = 0.045	Need bigger dataset for model training
2024	Multi-view spoofing detection approach focusing on the audio-text-emotion correlation^29^	ASVspoof-2021 (LA, DF) ASVspoof-2015 Fake or Real FakeAVCeleb In the wild	2021 LA EER = 2.00, min-tDCF = 0.2408 2021 DF, EER = 3.82 ASV-15, EER = 0.12 Fake or Real, EER = 4.55 FakeAVCeleb, EER = 8.03 In the wild, EER = 9.50_	Removing silent audio reduces performance Computationally complex
2023	Prosodic and pronunciation features-based spoofing detection approach^30^	ASVspoof-2019 (LA) ASVspoof-2015 VCC202 In the wild ADD2020 track2	2019 LA, EER = 1.58 ASVspoof-2015, EER = 3.08 VCC202, EER = 14.76 In the wild, EER = 36.84 ADD2020 track2, EER = 29.53	Effective for TTS-generated spoofed audio, however struggle with cloned audio
2023	Physiological-physical feature fusion approach^31^	ASVspoof-2019 (LA)	EER = 2.82, min-tDCF = 0.074	
2024	Spectra-temporal fusion-based approach^18^	ASVspoof-2019 (LA, PA) ASVspoof-2021 (LA, DF) VSDC Partial spoofs In the wild	2019 LA, EER = 0.22 2019 PA, EER = 0.52 2021 LA, EER = 3.50 2021 DF, EER = 3.20 VSDC, EER = 0.80 Partial spoofs, EER = 5.90 In the wild, EER = 0.30	Performance is unknown for algorithm-wise spoofing attacks
2025	Time and frequency domain STFT features fusion with self-attention and ResNet classifier^32^	ASVspoof-2019 (LA, PA) ASVspoof-2021 (PA, DF)	2019 LA, EER = 4.49 2019 PA, EER = 6.37 2021 PA, EER = 9.67 2021 DF, EER = 8.94	Results need to improve Performance for algorithm-wise spoofing remains unknown
2D Spectrogram Image-Based Approaches				
2023	Edge-mapped spectrograms and SVM classifier^20^	ASVspoof-2017 ASVspoof-2019 (LA, PA)	2017, EER = 19.89 2019 LA EER = 5.00, min-tDCF = 0.1177 2019 PA EER = 3.38, min-tDCF = 0.089	Less effective than deep learning approaches for detecting spoofed audio
2023	AVFakeNet^23^	ASVspoof-2019 (LA)	EER = 0.13	Performance is yet to be tested for ASVspoof-2021 dataset
2023	Joint training of audio enhancement and spoofing models^19^	ASVspoof-2019 (LA) FAD dataset	2019 LA, EER = 8.06 FAD dataset, EER = 1.06	Generalizability is uncertain Evaluation against TTS, VC, and deepfake-based attacks is un-explored
2024	ViT-based approach^22^	ASVspoof-2021 (LA)	EER = 4.74	
2024	VGGish framework incorporation attention block^21^	ASVspoof-2019 (LA, PA) ASVspoof-2021 (DF)	2019 LA EER = 0.07, min-tDCF = 0.03 2019 PA EER = 0.52, min-tDCF = 0.05 2021 DF, EER = 0.78	Unknown performance for partially spoofed audio samples and adversarial attacks
2025	MelCochleaGram spectrograms + ResNet50 classifier^34^	DECRO VSDC	DECRO, EER = 1.2 VSDC, EER = 1.4	Performance is unknown for unseen spoofing attacks
Other Spoofed Audio Detection Frameworks				
2021	Spoofprint framework^35^	ASVspoof-2019 (LA)	EER = 0.62	Dependency on speaker’s enrolment and lacks cross-corpora validation
2021	Global modulation features-based model^36^	ASVspoof-2019 (LA)	EER = 6.325, min-tDCF = 0.1387	Performance on ASV-2021 is unexplored
2022	StateNet—RawNet2-based framework learning spatio-temporal features^14^	ASVspoof-2019 (LA) For-Norm Dataset In the wild	2019 LA EER = 2.45, min-tDCF = 0.062 For-Norm Dataset EER = 0.81 In the wild EER = 0.19	Performance is unknown for partially spoofed audio samples and adversarial attacks
2022	Conformer-based framework^37^	ASVspoof-2019 (LA)	EER = 7.517 min-tDCF = 0.1561	The results need to be further improved
2023	Conformer Feature pyramid^38^	ASVspoof-2019 (LA)	EER = 1.65 min-tDCF = 0.047	Not evaluated for deepfake attacks
2023	Knowledge amalgamation–based framework^39^	ASVspoof-2019 (LA, PA)	2019 LA EER = 2.39, min-tDCF = 0.067 2019 PA EER = 1.97, min-tDCF = 0.059	Performance for the deepfake attacks is uncertain
2023	RawNet2 with the vocoder identification module^15^	ASVspoof-2019 (LA) Wavefake LibriSeVoc	2019 LA, EER = 4.54 Wavefake, EER = 0.19 LibriSeVoc, EER = 0.13	The generalization capability needs to be improved
2024	RawNet2-based meta-learning framework^16^	ASVspoof-2019 (LA)	EER = 0.87 min-tDCF = 0.0277	Not evaluated on PA and deepfake attacks
2025	Attack-specific expert and adaptive expert fusion approach^40^	ASVspoof-2019 (LA)	EER = 1.10	Performance is yet to be explored for ASVspoof-2021 dataset
2025	DeepLASD framework^41^	ASVspoof-2019 (LA) ASVspoof-2021 (LA)	2019 LA EER = 5.27, min-tDCF = 0.1216 2021 LA EER = 12.76, min-tDCF = 0.4250	Generalization needs enhancement ASVspoof-2021 DF evaluation is unknown
2025	Latent space refinement and augmentation-based approach^42^	ASVspoof-2019 (LA) ASVspoof-2021 (LA, DF) In the wild	2019 LA, EER = 0.15 2021 LA, EER = 1.19 2021 DF, EER = 2.43 In the wild, EER = 5.92	The performance is unknown for the spoofing adversarial attacks

### Conventional approaches for spoofed audio detection

Traditional approaches for audio spoofing detection comprise two modules: (i) a front-end feature extractor that extracts dynamic attributes from the audio via handcrafted features, and (ii) a back-end classifier that learns the features and discriminates between spoofed and bonafide audio. Such approaches have been commonly employed for audio spoofing detection tasks. In^3^, the front-end descriptor extracted the features based on the combination of GTCC, MFCC, spectral centroid, and spectral flux. The BiLSTM classifier was used to classify the spoofed audio. Likewise, Tuba et al.^4^ presented the framework that captured the dynamic traits of voice samples using the ELTP-LFCC features, with the BiLSTM classifier. ATP-GTCC was introduced in^5^ to capture the spoofed audio’s harmonic distortions and unnatural patterns. The extracted features were then used to train the support vector machine (SVM) classifier. In^6^, a spoofing detection approach was introduced that employed GTCC spectrogram features as the front-end and utilized a pre-trained ResNet50 model as a back-end to classify the extracted features. These conventional methods are computationally complex and have not been evaluated for the cross-corpora scenario; thus, the generalizability aspect of such approaches is unknown. Additionally, hand-crafted feature-based approaches are incapable of capturing complex variations of speech signals, and thus may not be able to perform well in the case of unseen spoofing attacks.

### One-class learning-based methods

One-class learning has been immensely applied for anomaly detection and involves the classification methods that capture and set boundaries around the positive class distribution. All the samples outside the distribution boundary are considered negative class samples. The main objective of one-class classification is to improve the generalizability of the anti-spoofing models to enable them to identify unknown spoofing attacks effectively. Zhang et al.^7^ introduced the one-class softmax that learned the bonafide speech embedding, whereas the spoofing data was at an angular margin from the bonafide data. In^8^, multi-conditional training, various mini-batching, and data-feeding techniques were discovered for the one-class learning of the spoofing detection models. Zhang et al.^9^ presented a method that incorporated autoencoders and support vector data description to learn bonafide speech features. The methods^7,9^ do not consider the bonafide speech diversity that exists due to various speakers, recording environments, and channels. Therefore, in^10^, the speaker attractor multi-center one-class learning (SAMO) method was introduced that constructed multiple clusters of bonafide speech based on the speaker’s identity. A knowledge distillation-based one-class learning framework was presented in^12^ to learn the distribution of bonafide speech and improve generalizability. The teacher model learns the distribution of both spoofed and bonafide data; thus, this method^12^ is not solely based on one-class embedding learning. Ren et al.^13^ introduced a lightweight framework implementing dispersion one-class softmax loss along with a knowledge distillation technique. To improve the spoofing detection accuracy and generalizability, a spectro-temporal model was introduced in^27^ that utilized the F0 subband to capture subtle features along with the OC-softmax incorporating KoLeo regularizer. One-class learning methods have achieved sustainable performance; however, their vulnerability against adversarial attacks should be addressed.

### Multi-view features fusion-based deep learning frameworks

For effective and generalized fake audio detection, many approaches have been introduced in recent literature that focus on feature fusion and finding the correlation between features captured from multiple dimensions. For instance, Doan et al.^28^ presented the breathing talking silence encoder (BTS-E) framework, capturing the correlation between silence, talking, and breathing sounds for the spoofed audio detection. The spoofing aware transformer network (SpoTNet) introduced in^17^ was comprised of a spoofing feature extraction module, a logical spoofing transformer encoder (LSTE), and a multilayer spoofing classifier. To effectively train the LSTE module incorporating the transformer encoder, a large amount of training data is required. Junyan et al.^29^ introduced a spoofing detection approach that extracted multi-view features by focusing on the audio-text-emotion correlation. The removal of silent audio parts results in a performance decline, indicating the model overfits to the silent audio parts. Also, textual feature extraction is computationally complex. To detect different spoofing attacks,^30^ presented a generalized framework that captured the multi-view features such as phoneme duration and pronunciation features. The features were fused using the attention mechanism and then classified through a deep back-end classifier. The approach introduced in^31^ focused on the fusion of the physiological and speech features using the attention-based feature fusion method. Such approaches^30,31^ can work effectively for the spoofed audio generated through TTS but not for the cloned audio. Lian et al.^32^ demonstrated an approach that fused multimodal time- and frequency-domain features through a self-attention mechanism and performed classification using a ResNet50 classifier. Mostly, multi-view features have shown effective performance while detecting spoofing attacks; however, such methods that employ transformer-based architectures require a large amount of data for training. Additionally, very little attention is given to the detection of audio deepfakes attacks.

### 2D spectrogram image-based approaches

Existing approaches have also utilized 2D spectrogram images of the audio samples for accurately identifying the synthetic voice samples. The approach that involved the joint training of speech enhancement (U-Net) and anti-spoofing (LCNN and ResNet) models was demonstrated in^19^. This approach was evaluated on the noisy samples of ASVspoof-2019 LA and FAD datasets and achieved enhanced performance for the scenarios of low signal-to-noise ratio. To detect the spoofed audio, an approach that carried out the texture analysis of the edge-mapped audio spectrogram was introduced in^20^, but this approach is less effective for spoofed audio detection, as the reported results did not outperform the existing DL methods. Kanwal et al.^21^ presented the VGGish network that incorporated a convolutional block attention module for effective feature representation of mel-spectrogram images of the audio. Goel et al.^22^ presented a vision transformer-based framework where the self-supervised audio spectrogram transformer, basically a vision transformer (pre-trained on LibriSpeech and AudioSet datasets), was employed as the backbone. Cochleagram representations have also been used for speech processing, including clinically relevant applications such as cochlear implants, due to the ability to capture temporal and spectral information^33^. For the spoofing detection, ^34^ introduced an approach utilizing MelCochleaGram (MCG) spectrogram, a combination of Mel Spectrogram and Cochleagram, providing the visualization of frequencies over time. These MCG spectrograms were classified using a pre-trained ResNet50. The approaches^19,20,22,34^ are not assessed for the unknown TTS, VC, and deepfake-based spoofing attacks. More research is required to enhance the robustness and effectiveness of spectrogram image-based approaches while detecting audio spoofing attacks.

### Other spoofed audio detection frameworks

Several DL-based approaches have been introduced in the literature to enhance the generalization aptitude for the detection of spoofing attacks. To improve the generalizability, the framework, namely spoofprint, was introduced in^35^, which consisted of an enrolment and verification phase to detect the spoofing attacks. The attack-specific and cross-corpora evaluation is required to validate the generalizability. Additionally, spoofprint constraints the enrolment of the speaker for the detection task. Gao et al.^36^ presented a generalized approach based on global modulation features capturing long-term spatio-temporal information. To learn the local and long-term global feature dependencies, conformers have been introduced that incorporate a convolutional neural network (CNN) and transformer architectures. Rosello et al.^37^ applied the conformer-based approach for audio spoofing detection and evaluated it on the ASVspoof-2019 LA dataset. The method^37^ has attained encouraging results that need to be further improved. The feature pyramid module, along with a conformer block capturing the global context and local details, was introduced in^38^. A feature pyramid module was adopted to aggregate the output of each conformer block and thus learn the discriminative features of the spoofed and real audio samples. The approach introduced in^39^ employed two amalgamation mechanisms, namely structural and feature amalgamation, that enabled the student model to capture the semantic and structural knowledge from the teacher models. Adversarial learning, along with feature matching loss, was utilized to align feature embeddings and enforce structural consistency. Sun et al.^15^ demonstrated a multi-task framework consisting of a RawNet2-based model that detected the vocoder artifacts present in the spoofed audio samples. The generalization capability of the approach^15^ needs to be improved. A meta-learning framework was presented in^16^ along with the adversarial learning approach and RawNet2-based feature encoder incorporating a simple attention module for effective feature extraction. To improve the robustness against noise and channel compression, a framework, namely Adaptive Mixture Lowrank Experts (AUMLET), was introduced in^40^. DeepLASD framework based on RawNet with the incorporation of GELU activation function in the residual block was presented in^41^. The generalization ability of this approach^41^ needs to be further validated. To enhance the generalizability of audio spoofing detection approaches, Wen et al.^42^ introduced the strategy of latent space augmentation and refinement and demonstrated their effectiveness via achieving competitive results.

The literature review demonstrated the effectiveness of the existing anti-spoofing methods; however, there are still challenges that need to be addressed to make the models effective for real-world applications. For instance, DL-based spoofing detection methods, including one-class learning-based, multi-view features-based, and 2D spectrogram-based models, are vulnerable to adversarial attacks in real-world scenarios. The effective performance of the spoofing detection models against the partially spoofed audio samples needs to be tackled. Additionally, the effectiveness against the detection of unknown spoofing attacks, including the audio deepfakes attacks and cross-corpora evaluation, is essential to demonstrate the models’ generalizability and combat the real-world voice spoofing attacks.

## Proposed methodology

**Figure 2:**
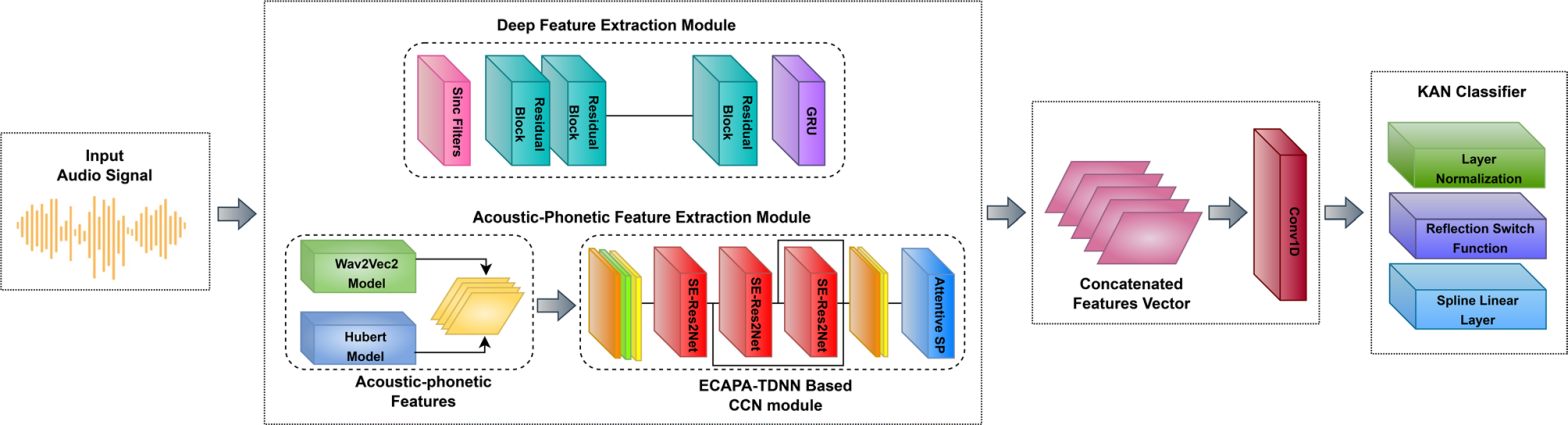
Architecture diagram of phonetic-DeepKANet.

This section describes the architectural details of the proposed framework, namely Phonetic-DeepKANet. The detailed diagram of the proposed methodology is presented in Fig. 2. The proposed PDK-Net processes the raw audio for the detection of spoofed utterances. Specifically, PDK-Net is a dual-modality network comprising TransRawNet and CNN modules to capture deep and multi-view acoustic–phonetic features. In our proposed framework, TransRawNet extracted deep representations, whereas acoustic–phonetic embeddings are obtained using HuBERT and wav2vec, further processed through ECAPA-TDNN having the ability to model temporal dependencies. In general, deep features complemented with multi-view features enable the proposed model to capture the spoofed speech artifacts that appear at multiple representation levels, including signal, phonetic, and spectral. Thus, these combined features capture the diverse patterns and comprehensive representations of audio signals, leveraging the strengths of both modalities, thus aiding in improving the performance. The extracted features are concatenated, then encoded using a single convolutional layer, and finally passed to the Kolmogorov Arnold Network to effectively model the relationships among heterogeneous feature representations. KAN classifier performs adaptive, feature-wise functional transformations that enable more expressive modeling of complex feature distributions and improved generalization. The hierarchical structure of KAN enables feature learning at multiple resolutions, allowing the model to progressively emphasize the fine-grained artifacts relevant to spoofing detection. The key components of the proposed framework are the deep feature extraction module M_DF_, the acoustic–phonetic feature extraction module M_APF_, and the KAN classifier C_KAN_. The proposed network can be expressed as:

1 $$F_{DF} = M_{DF} \left( {X_{i} } \right)$$

2 $$F_{APF} = M_{APF} \left( {X_{i} } \right)$$

3 $$F_{con} = Concat\left( {F_{DF} , F_{APF} } \right)$$

4 $$F = Conv\left( {F_{con} } \right)$$

5 $$O_{pred} = C_{KAN} \left( F \right)$$

where $X_{i}$ refers to the input audio signal, $F_{DF}$, $F_{APF}$, $F_{con}$, and $F$ represents the extracted deep features, acoustic–phonetic features, concatenated features, and the final features vector, respectively. $M_{DF} ,$ $M_{APF}$, and $C_{KAN}$ corresponds to the deep features extraction module, acoustic–phonetic features extraction modules, and KAN classifier module, respectively. $O_{pred}$ indicates the final output of the proposed PDK-Net. The details of each module are provided in the subsequent subsections.

### Deep feature extraction module

**Fig. 3:**
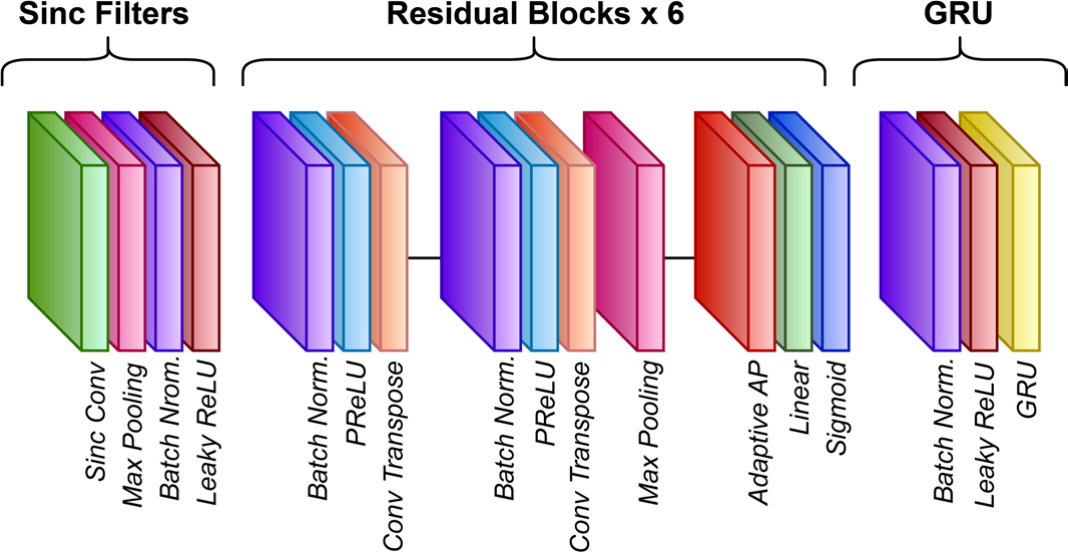
Architectural detail of TransRawNet.

The deep feature extraction module is employed to enable the framework to learn the deep representation of input raw audio samples. The purpose of the deep features is to capture complex hierarchical representations, including phase information and temporal patterns, that aid in the detection of subtle variations between spoofed and real utterances. For this, we introduced TransRawNet, and the layers included in the architecture are presented in Fig. 3. The first layer is the Sinc filter block, whereas the higher layers include residual blocks and gated recurrent units (GRU). Sinc Conv is the constrained convolutional layer in the Sinc filters, instead of all coefficient filters, it only learns the meaningful bandpass filters parameterized by the Sinc function. In Residual blocks, PReLU activation function and transpose convolution are employed, which aid in the enhancement of model performance for the detection of spoofed audio. The transpose convolution layer performs the convolution operation with an upsampling mechanism using the fractional strides; thus, the output feature map has increased spatial dimensions. This characteristic of the transpose convolution layer aids in recovering spatial information loss during the downsampling operation. Thus, enabling the model to generate detailed, information-rich, and high-resolution feature vectors, leading to an improvement in performance. PReLU activation function reduces the dying ReLU problem via enabling the model to adaptively learn the optimal negative slope. With this incorporated activation function, the model can better capture the subtle features from the diverse audio data and improve the generalization on the unseen data. Additive average pooling, linear, and sigmoid layers act as an attention mechanism, combining the additive and multiplicative scaling approach to capture the more discriminative representation of real and spoofed audio. Finally, a GRU layer is employed to generate utterance-level embeddings via aggregating frame-level representations and captures the speech dynamics across the utterances. In general, TR-Net processes the raw audio and generates the deep feature vector containing the discriminative, subtle, and high-resolution feature representation of the given audio sample.

### Acoustic–phonetic feature extraction module

The acoustic–phonetic feature extraction module comprises two main components: multi-view feature extraction and a CNN module whose inputs are the multi-view features. The aim of incorporating the multi-view features is to improve the generalization aptitude of the PDK-Net via learning the multiple aspects of the audio waveform. In our proposed framework, multi-view features capture the generalized feature representations from phoneme duration and wav2vec dimensions. Wave2vec features represent phonetic patterns and are robust for noisy environments and degraded audio samples. However, features extracted utilizing the Hidden Unit BERT (HuBERT) model^43^ capture the abrupt phoneme transitions useful for identifying temporal phoneme anomalies in spoofed audio samples. These feature representations are then passed to the CNN module based on ECAPA-TDNN^44^, for further processing. Further details are given in the following subsections.

#### Acoustic–phonetic features

To capture the phoneme duration features, the audio signal is encoded using the HuBERT model pre-trained on 960h of the LibriSpeech corpus. The resultant encoded vector is considered a speech phoneme duration vector. Likewise, the pre-trained wav2vec model^45^ is utilized for the extraction of diverse and robust speech representations from audio signals. The use of transformer architecture in the wav2vec model aids in capturing long-term dependencies. The wav2vec model outputs 1024 dimensions, which is reduced to 128, using a fully connected linear layer, to reduce computational complexity. The HuBERT model captures the high-level phonetic representations that are not sensitive to speaker variations. However, the wave2vec dimensions comprise low-level features, including acoustic and phonetic features, along with some speaker-specific attributes. These features are then concatenated along feature dimensions, resulting in multi-view rich acoustic and phonetic representations. These representations capture the generalized speech patterns and local and global context, leading to improved performance for the detection of the spoofed audio.

#### ECAPA-TDNN-based CNN module

ECAPA-TDNN is the extended version of the time delay neural network, with enhancements such as SE-Res2Net blocks, an attentive statistical pooling layer, and multi-layer feature aggregation. This network is capable of effectively capturing the subtle features of real and spoofed audio via utilizing its multi-scale feature aggregation and attention mechanisms. The extracted multi-view acoustic-phonetic features passed to the ECAPA-TDNN module enable it to learn a comprehensive representation space, resulting in generalizable embeddings, thus enabling the framework to generalize well to unseen audio samples. The statistical pooling layer outputs the utterance-level representations, which are then concatenated with the utterance-level embeddings from the deep feature extraction module. The concatenated representations are then encoded utilizing the convolution layer. The encoded representations are then passed to the KAN for the classification of real and spoofed audio.

### KAN Classifier

Kolmogorov Arnold Network is utilized as a classifier in our proposed framework, PDK-Net, due to its ability to model complex interactions between heterogeneous feature representations through learnable univariate functions. It specifically learns adaptive univariate functions along network edges, enabling more flexible functional approximation. KAN is based on the Kolmogorov-Arnold representation theorem, stating that any multivariate function can be represented as a sum of univariate functions. It implements a hierarchical structure, enabling it to model complex multivariate functions via decomposition into simpler univariate functions, thus making the learning process more efficient. Mathematically, it can be represented as:

6 $$C\left( F \right) = C \left( {F_{1} , F_{2} , F_{3} , \ldots , F_{m} } \right) = \mathop \sum \limits_{l = 1}^{2m + 1} {\Phi}_{l} \mathop \sum \limits_{k = 1}^{m} \varphi_{l,k} \left( {F_{k} } \right)$$

where $F_{k}$ represents the input features, $\varphi_{l,k}$ is a univariate function applied to the input features, and ${\Phi}_{l}$ is a univariate function applied to the output of $\varphi_{l,k}$.

The incorporated KAN classifier is mainly composed of (i) layer normalization (LN) to standardize the input representations, (ii) reflection switch function (RSF) that generates non-linear spline transformations, and (iii) spline linear (SL) layer applying the linear transformation to the transformed spline basis. The incorporated KAN classifier architecture can be represented as:

7 $$F_{norm} = LN \left( F \right)$$

8 $$F_{RSF} = RSF \left( {F_{norm} } \right)$$

9 $$O = W \cdot F_{RSF} + b$$

where $F$ indicates the concatenated feature vector, $F_{norm}$ is the normalized feature vector obtained through the normalization layer $LN$, and $F_{RSF}$ represents the feature vector after applying the radial switch function transformation. $O$ corresponds to the output obtained when the learned weight matrix $W$ is applied to $F_{RSF}$ in the spline linear transformation.

These layers in the KAN classifier learn data-adaptive univariate functions for each feature, enabling explicit modeling of feature-level functional relationships rather than relying on fixed activation functions as in conventional MLPs. These characteristics of RSF and SL layers allow the framework to effectively capture feature-wise functional dependencies and fine-grained variations, such as subtle patterns introduced by the speech synthesis methods. Thus, enabling the framework to estimate complex decision boundaries and improving the generalization over unseen spoofing attacks. In general, the KAN enables compact and powerful functional representation capable of capturing higher-order feature interactions, in contrast to Transformer-based classifiers or large-margin softmax variants, which primarily rely on attention mechanisms or linear projections within the embedding space. Finally, there is a fully connected layer to produce the final prediction and classify the given audio samples as either real or spoofed.

## Experimental results

This section provides the details of the conducted experiments and results analysis to demonstrate the effectiveness of the proposed PDK-Net framework. The datasets used for the evaluation are also described in this section. To show the performance of the proposed method, we have utilized the standard evaluation metrics, namely the minimum tandem detection cost function (min-tDCF) and equal error rate (EER)^46^. Min-tDCF corresponds to the combined performance of the spoofing countermeasure (CM) and ASV system; however, EER represents the spoofing CM point, where the false positive and negative rates are equal.

### Dataset

To analyze the performance of the proposed method, we have utilized the ASVspoof-2019 LA^47^, ASVspoof-2021 LA and DF^46^, and partial spoof^48^ datasets. Moreover, we also created a novel Arabic audio spoofing dataset to evaluate the performance of the proposed PDK-Net for Arabic audio spoofing detection. The details of the datasets are provided in the subsequent subsections.

#### ASVspoof-2019 dataset

ASVspoof-2019 LA dataset is comprised of training, development, and evaluation subsets having 25,380, 24,844, and 71,237 audio samples, respectively. The spoofing utterances are generated using the 17 different TTS and VC algorithms (A1-A17), among which six algorithms (A1-A6) are known, and eleven algorithms (A7-A19, except A16 and A19) are unknown spoofing systems.

#### ASVspoof-2021 dataset

The ASVspoof-2021 dataset is composed of the evaluation subset only, whereas the ASVspoof-2021 LA and DF subsets have approximately 148k and 612K utterances, respectively. ASVspoof-2021 LA dataset is more challenging as it introduced unknown channel variations to the audio samples. The speech utterances contain the transmission and encoding artifacts introduced by telephone network systems and voice-over-Internet-Protocol systems. ASVspoof-2021 DF dataset comprises spoofed audios generated through 100 different spoofing attack algorithms. It contains the spoofed and bonafide samples that undergo different codec compressions.

#### Partial spoof dataset

The partial spoof dataset comprises partially spoofed audio generated utilizing the ASVspoof-2019 LA dataset. The dataset is generated by randomly substituting bonafide segments into spoofed audio and vice versa. The number of utterances is the same as in the ASVspoof-2019 LA dataset.

#### Arabic audio spoofing dataset

In this study, we developed an Arabic audio spoofing dataset^49^ to support the spoofed speech detection of underrepresented languages. The dataset mainly comprises bonafide and spoofed audio samples generated using voice conversion techniques. The bonafide audio samples in the Arabic language are part of the MediaSpeech dataset^50^ and are obtained from an open speech and language resources repository^51^, presenting 10h of speech. The bonafide speech originates from publicly available broadcast media sources and includes speech samples from multiple speakers under realistic recording conditions. Specifically, the bonafide utterances are the speech segments extracted from the videos on official YouTube media channels (Al Arabiya, France 24 Arabic, BBC News Arabic), thus depicting real-world multi-speaker audio data. Detailed information regarding the collection of Arabic bonafide utterances is provided in the MediaSpeech dataset paper^50^. To preserve the multi-speaker attribute, prosodic and conversational variations of the bonafide samples, we choose voice conversion techniques for generating spoofed utterances. Specifically, the spoofed samples are generated using five voice conversion algorithms, namely, SeedVC^52^, DiffHierVC^53^, FreeVC^54^, HierSpeechVC^55^, and KNNVC^56^. The bonafide data is first split into train, development, and evaluation sets. To generate the spoofed samples, one utterance was considered as the target, while five randomly selected audio samples were used as source audio, one at a time. The generated spoofed samples mimic the conversation style and pronunciation of the Arabic speakers, thus increasing the complexity of the dataset. We used the 2505 bonafide samples to generate the 62,625 spoofed samples. Following the design philosophy of ASVspoof challenges, the dataset is split into train, development, and evaluation sets, where the evaluation set is much larger than the train and development sets. The train and development sets encompass a total of 16,276 audio samples, having 626 bonafide and 15,650 spoofed utterances. However, the evaluation set comprises 32,578 audio waveforms with 1,253 bonafide and 31,325 spoofed samples.

### Experimental setup

The proposed framework is implemented using the PyTorch Python framework. The overall framework contains approximately 24M parameters and requires about 3.25 GMACS per forward pass. The proposed model is trained for 30 epochs with a batch size of 16, using binary cross-entropy loss and Adam optimizer with an initial learning rate of $1 \times 10^{ - 4}$. To dynamically adjust the learning rate during training, the ReduceLROnPlateau learning rate scheduler was employed with a reduction factor of 0.8 and patience of 2. All the experiments were conducted on a high-performance computing machine with 32 GB RAM and a 24GB NVIDIA 3090 GPU.

### Performance evaluation

This experiment is conducted to evaluate the performance of the proposed PDK-Net on Arabic and English audio spoofing samples. Particularly, to evaluate the performance of the proposed method for Arabic audio spoofing, we conducted an experiment utilizing our own introduced Arabic spoofing dataset. For this, PDK-Net is trained on a combined training and development set; however, the evaluation is done on the evaluation set of the Arabic spoofing dataset, results are presented in Table 2. The PDK-Net attained an EER of 8.06 for the Arabic spoofing dataset, indicating the proposed model’s potential for the detection of Arabic spoofing samples.

**Table 2:** Evaluation on Arabic and English (ASVspoof-2019 LA) audio spoofing datasets.

Datasets		min-tDCF	EER (%)
Arabic Spoofing Dataset		–	8.06
ASVspoof-2019 LA dataset	Overall LA attacks	0.09	4.55
	VC attacks	0.21	8.80
	TTS attacks	0.02	0.80

The performance of the model for the English language is evaluated using the ASVspoof-2019 LA dataset. For this, we trained the PDK-Net on a combined training and development set. The trained framework is then evaluated on the evaluation set of the ASVspoof-2019 LA dataset, and the results are shown in Table 2. On the whole LA dataset, the proposed framework attained min-tDCF and EER of 0.09 and 4.55, respectively. This indicates the effectiveness of the proposed method for detecting LA attacks. Specifically, the extracted combined deep and multi-view features empower the proposed PDK-Net to accurately identify the known and unknown LA attacks. The performance is further evaluated through multiple experiments, including evaluation on VC attacks, evaluation on TTS attacks, and algorithm-wise evaluation. The details of the experiments are provided in the subsequent subsections.

#### Evaluation on voice conversion attacks

This experiment is designed to evaluate the performance of our method for voice conversion attacks. Voice conversion attacks involve the generation of fake audio from the reference real audio samples and retain the prosodic features of the voice. Thus, enabling the algorithm to generate realistic fake voices and making the detection more challenging. The training and development set of ASVspoof-2019 LA dataset contains VC attack samples generated via two algorithms (A05 and A06), whereas the evaluation set comprises the VC samples created through three types of algorithms (A17, A18, A19). The proposed method is trained on the VC samples from the training and development set and then evaluated for the VC attack samples of the evaluation set. The results in terms of min-tDCF and EER are reported in Table 2. The attained min-tDCF of 0.21 indicates the effectiveness of the proposed method for the detection of VC attacks. This is mainly due to the ability of multi-view acoustic–phonetic features to identify the subtle prosodic inconsistencies and unnatural phoneme transitions in the voice-cloned utterances. Additionally, the detection of artifacts introduced due to the generation process of the cloned voices, using the deep features from TR-Net, further aids in the accurate detection of the voice cloning attacks.

#### Evaluation on text-to-speech attack

To assess the proposed method’s effectiveness for the detection of text-to-speech attacks, an experiment is conducted on the TTS attack samples of the ASVspoof-2019 LA dataset. Text-to-speech synthesis involves the generation of realistic synthetic audio from the given text, using the predefined voices based on the utilized model. The evaluation set of the dataset includes TTS samples produced via 7 distinct attacks (A7–A12, A16), whereas the training and development set of the dataset consists of TTS samples generated through 4 distinct algorithms (A1–A4). For the experiment purpose, the framework is trained on the TTS samples from the training and development sets. The trained PDK-Net is then assessed on the TTS samples from the evaluation set of the ASVspoof-2019 LA dataset. The attained results on TTS attack samples are shown in Table 2. Min-tDCF of 0.02 indicates that the proposed method remarkably captures the distinct features of real and TTS-generated voices. Fake audio samples generated using TTS algorithms generally lack the prosodic characteristics of real voices and have smooth transitions between phonemes and unnatural silences. The combined deep and acoustic–phonetic features learn high- and low-level anomalies among audio samples and effectively capture such variations between real and spoofed utterances, thus improving the performance. Concisely, the detection results reveal that synthesized artifacts are effectively identified by the proposed PDK-Net during the detection of the TTS attacks.

#### Evaluation on algorithm-wise attacks

To assess the proposed PDK-Net effectiveness against each algorithm used to generate VC and TTS attack samples involved in the ASVspoof-2019 LA dataset, an experiment is designed where the method is evaluated for the different known and unknown attacks. The evaluation set of ASVspoof-2019 LA dataset comprises five unknown (A7–A12) and one known (A16) TTS attacks, two unknown (A17, A18) and one known (A19) VC attacks, and three unknown TTS-VC (A13–A15) attack samples. For the experiment, the protocol for training the PDK-Net is the same as in "Performance evaluation" section. However, the trained framework is separately evaluated for each attack algorithm, and results are reported in Table 3. From the results, it can be observed that min-tDCF below 0.1 is achieved for most of the attacks except for A17 and A18. For A17, a min-tDCF of 0.176 is attained, whereas the min-tDCF is 0.546 for A18, which is competent for the detection of such unknown attacks. Both (A17 and A18) are the VC attacks based on the acoustic encoder and waveform filtering models, which are more challenging attacks. Along with the precise detection of known attacks, the learned generalized multi-view and deep acoustic–phonetic features, when classified using the KAN, also aid in the effective detection of challenging unknown spoofing attacks.

**Table 3:** Algorithm-wise evaluation on ASVspoof-2019 LA dataset.

Metrics	A07	A08	A09	A10	A11	A12	A13	A14	A15	A16	A17	A18	A19
EER (%)	0.832	2.380	0.268	1.205	0.693	0.978	0.105	0.268	0.937	0.750	5.639	17.523	1.223
min-tDCF	0.026	0.058	0.008	0.039	0.022	0.031	0.003	0.008	0.029	0.024	0.176	0.526	0.041

### Evaluation on statistical and adversarial attacks

To assess the aptitude of the proposed method for the detection of perturbed audio samples, we conducted a two-stage experiment, where in the first stage, the PDK-Net is evaluated against statistical attacks. However, in the second stage, the framework’s robustness is assessed against white-box and black-box adversarial attacks. For the statistical attacks, we individually introduced the Gaussian Noise, Pitch Shift, Time Stretch, and Shift as perturbations on the utterances of the evaluation set of ASVspoof-2019 LA dataset. However, we generated adversarial attack samples of the ASVspoof-2019 LA evaluation set, using Fast Gradient Sign Method (FGSM)^57^, Projected Gradient Descent (PGD)^58^, Carlini & Wagner (C&W)^59^, DeepFool^60^, and HopSkipJump^61^ attacks. This set of attacks ensures the comprehensive assessment of the proposed method under varying attack strengths and adversarial strategies. To reflect the realistic adversarial scenario, where the attacker has limited knowledge of the target model, adversarial perturbations are crafted using a surrogate model (RawNet). Afterward, the PDK-Net trained on the ASVspoof-2019 LA dataset is utilized to test the perturbed samples generated through statistical and adversarial attacks. The results are reported in Table 4. It can be noticed that the performance of the framework decreases for the perturbed samples compared to the performance on the clean LA dataset, demonstrating the impact of small perturbations on the model’s effectiveness. Among the applied statistical perturbations, the proposed method is more robust towards Gaussian Noise, attaining the min-tDCF of 0.117. Whereas, for Pitch Shift, Time Stretch, and Shift perturbations, the achieved min-tDCF is above 0.33. This indicates the robustness of our method for the detection of noisy utterances, since during training, the framework considered the noisy variants of audio samples. For the adversarial attacks, the min-tDCF remains nearly constant across all adversarial attacks, ranging from 0.197 to 0.199. However, the variation in EER can be observed, reflecting that the adversarial perturbations mainly affect the classification boundary instead of cost-sensitive decision performance. In terms of EER, the PDK-Net demonstrates reasonable performance under HopSkipJump, DeepFool, and C&W attacks, highlighting the model’s robustness against black-box, minimal perturbation, and optimization-based attacks. Whereas, under FGSM and PGD attacks, the performance of the proposed framework degrades, indicating the sensitivity toward gradient-based perturbations. Concisely, the PDK-Net performance is satisfactory on the perturbed samples, indicating the ability of the proposed approach to tackle attacks, including statistical, white-box, and black-box adversarial attacks.

**Table 4:** Evaluation on perturbed samples of ASVspoof-2019 LA dataset.

	min-tDCF	EER (%)
Statistical Attacks		
Gaussian Noise	0.117	4.607
Pitch Shift	0.338	16.302
Time Stretch	0.344	16.047
Shift	0.369	13.503
Adversarial Attacks		
FGSM	0.197	10.18
PGD	0.196	10.15
C&W	0.198	9.78
DeepFool	0.199	9.85
HopSkipJump	0.199	9.82

### Cross-corpora evaluation

To demonstrate the generalization aptitude of the proposed method, cross-corpora evaluation is conducted. For this, the PDK-Net trained on the ASVsoof-2019 LA dataset is evaluated on the ASVspoof-2021 LA and DF datasets. We also conducted a cross-corpora evaluation for the partial spoof dataset. The details of the cross-corpora experiments are provided in the following subsections.

#### Evaluation on ASVspoof-2021 LA dataset

For evaluating the performance of PDK-Net on the ASVspoof-2021 LA dataset, the training protocols are kept the same as in "Performance evaluation" section. We tested our method on the audio samples of ASVspoof-2021 LA dataset, and the results are demonstrated in Table 5. The proposed approach attained a min-tDCF of 0.144 on overall LA attacks of the ASVspoof-2021 dataset. *To the best of our knowledge, the achieved min-tDCF of 0.144 is the lowest among all the challenge participants of the ASVspoof-2021 LA dataset.* This demonstrates the great generalization aptitude of the proposed PDK-Net method for the detection of logical access attacks that contain channel variations and transmission artifacts. For the detailed analysis, we also designed experiments where the method’s generalizability is assessed for the VC attacks, TTS attacks, and algorithm-wise VC, TTS, and VC-TTS attacks.

**Table 5:** Evaluation on ASVspoof-2021 LA dataset.

ASVspoof-2021 LA dataset	min-tDCF	EER (%)
Overall LA attacks	0.144	6.113
VC attacks	0.308	13.082
TTS attacks	0.062	1.829

##### Evaluation on VC attacks

For assessing the generalizability of the proposed method for voice conversion attacks, an experiment is conducted to evaluate the method on VC samples of the ASVspoof-2021 LA dataset. Precisely, the PDK-Net trained on VC samples of ASVspoof-2019 LA dataset is utilized to assess the VC samples of ASVspoof-2021 LA dataset. The experimental protocol for training is the same as in "Evaluation on voice conversion attacks" section. The evaluation results in terms of min-tDCF and EER are reported in Table 5. For the VC attacks of the ASVspoof-2021 LA dataset, we have achieved a min-tDCF of 0.308, indicating a good detection of the synthetic speech used over the phone to impersonate individuals or to deceive ASV systems. The obtained results validate the generalization ability of the PDK-Net, specifically for the detection of cloned voices.

##### Evaluation on TTS attack

This experiment is performed to analyze the generalization aptitude of our method for TTS attacks. For this, our framework is trained on TTS samples of the ASVspoof-2019 LA dataset, whereas the trained PDK-Net is then evaluated on TTS samples of the ASVspoof-2021 LA dataset. For the framework training, the experimental protocol is the same as in "Evaluation on text-to-speech attack" section. The results attained on TTS attacks of ASVspoof-2021 LA are shown in Table 5. It can be observed that the obtained results on TTS attacks are better compared to the VC attacks, as the min-tDCF is 0.062, which shows an excellent generalization performance of our spoofing countermeasure. These results demonstrate the robustness of the proposed method over phone channel variations and better generalization aptitude for the voice samples generated through text-to-speech algorithms.

##### Evaluation on algorithm-wise attacks

This experiment is designed to investigate the generalization potential of the PDK-Net for standalone spoofing attack detection in a cross-corpora setting. In this experiment, the framework trained on the ASVspoof-2019 LA dataset is utilized for the evaluation of algorithm-wise attacks of the ASVspoof-2021 LA dataset. The results of the proposed method for each attack are reported in Table 6, in terms of EER and min-tDCF. Likewise, ASVspoof-2019 LA dataset, the PDK-Net attained the higher min-tDCF of 0.747 and 0.327 for the challenging A18 and A17 unknown attacks. For all the other attacks, min-tDCF is below 0.09, indicating the robustness of the proposed method for the detection of individual spoofing attacks involving diverse telephone codecs for transmission, such as a-law, G.722, OPUS, and GSM.

**Table 6:** Algorithm-wise evaluation on ASVspoof-2021 LA dataset.

	A07	A08	A09	A10	A11	A12	A13	A14	A15	A16	A17	A18	A19
EER (%)	1.652	3.336	0.621	2.296	1.267	1.443	0.183	0.635	1.760	1.166	10.214	25.188	2.538
min-tDCF	0.056	0.089	0.021	0.079	0.044	0.049	0.005	0.021	0.059	0.037	0.327	0.747	0.085

The proposed PDK-Net demonstrates competent generalization aptitude when evaluated on the unseen samples of the ASVspoof-2021 LA dataset. This is mainly due to the ability of the PDK-Net to learn robust, multi-view, and generalized features and the incorporation of the KAN classifier. It enables the proposed method to perform well while detecting the VC-attacked audio samples of distinct datasets, in cross-corpora scenarios. Additionally, the deep acoustic–phonetic features better capture the discrepancies of TTS-based synthesized utterances. Along with that, the ability of the KAN classifier to learn adaptive univariate functions, allowing it to capture structured and subtle variations, further enhances the generalization aptitude of the framework for the VC and TTS attacks, along with the algorithm-wise attacks, as validated through the attained results.

#### Evaluation on ASVspoof-2021 DF dataset

To demonstrate the generalization capability of our approach for deepfake attacks, the PDK-Net is evaluated on the ASVspoof-2021 DF dataset. In the ASVspoof-2021 DF dataset, diverse encoding and compression standards (MP3, OGG, ACC) are employed for the audio signals to represent the scenario of compression that occurs during the uploading of synthetic speech to social media platforms. For this experiment, the PDK-Net trained on the ASVspoof-2019 LA dataset is used for the evaluation on the ASVspoof-2021 DF dataset. The proposed method achieved an EER of 17.554% on the ASVspoof-2021 DF dataset, which is the third-best EER among all 33 participants of the ASVspoof-2021 challenge. The ability of the PDK-Net to focus on phase distortions, phonetic and temporal inconsistencies, and subtle unnatural artifacts enables it to discriminate between real and deepfake audio samples. Thus, signifies the robustness of the proposed method for the detection of compressed synthetic speech and the ability to detect fake audio uploaded on social media platforms to disseminate disinformation.

#### Cross-corpora evaluation of partial spoof dataset

The main goal of this experiment is to investigate the generalizability of the framework, trained on the fully spoofed dataset, for the detection of partially spoofed audio signals. For this purpose, we conducted experiments where the method trained on the ASVspoof-2019 LA dataset was evaluated using the audio samples of the partial spoof dataset. The spoofed utterances in the partial spoof dataset comprise both spoofed and bonafide segments, making it challenging for the framework to accurately distinguish between spoofed and bonafide samples. Our proposed PDK-Net has attained a min-tDCF of 0.320 for the detection of partially spoofed utterances in cross-corpora settings. The attained results are rational in the scenario of the distinct dataset. The GRU layer incorporated in TransRawNet enables the proposed framework to capture the temporal inconsistencies in the audio sequences, thus facilitating the detection of partially spoofed segments of the audio signal. Additionally, the multi-view features learning the spoofed artifacts and unnatural irregularities in utterances further aid the proposed PDK-Net to effectively detect the audio that is partially spoofed.

### Ablation study

The ablation study is conducted to analyze the impact of different features on the overall performance of the proposed framework. For this purpose, we employed multiple features along with the deep features extracted using TransRawNet. Particularly, we analyzed wavelet features, constant-Q transform (CQT) features, wav2vec + phoneme duration, and wav2vec + phoneme duration + spectral contrast features. The main goal of utilizing such features is to evaluate the effectiveness of complementing deep features with time and frequency representations, spectral information, and acoustic and phonetic features for the audio spoofing detection task. The comparison in terms of min-tDCF is reported in Table 7.

**Table 7:** Comparison of the different feature variations.

Features Variations	min-tDCF
Wav2vec + phoneme duration + deep features	0.090
Wav2vec + phoneme duration + spectral contrast + deep features	0.131
Wavelet features + deep features	0.190
CQT features + deep features	0.100

From the results, it can be depicted that the multi-view features from phoneme duration and wav2vec dimensions, along with the deep features, outperform all the other feature variants by achieving a min-tDCF of 0.09. The method employing CQT features complementing deep features is the second-best performer, whereas the lowest performance is achieved through wavelet and deep features. Moreover, the combination of spectral contrast with phoneme duration and wave2vec features is also not proven effective for detecting spoofed audio samples. These results reveal that the wav2vec + phoneme duration + deep features provide a comprehensive feature vector for the audio signal, leading it to outperform other feature combinations in the proposed framework, PDK-Net.

### Comparative analysis

This section represents the extensive comparative study that is conducted to demonstrate the effectiveness of our PDK-Net for the detection of spoofing attacks against state-of-the-art approaches. Specifically, the performance of the proposed method is compared with the baseline models for the ASVspoof and Arabic spoofing datasets. Additionally, the comparison with existing approaches and challenge participants of the ASVspoof-2021 dataset is also conducted. The comparison can be found in the subsequent subsections.

#### Comparison with baseline models

The goal of this comparative analysis is to show the effectiveness of the PDK-Net against the baseline methods for the detection of spoofing attacks in English and Arabic languages. Specifically, we compared PDK-Net performance with baselines LFCC-GMM, CQCC-GMM, LFCC-LCNN, and RawNet2 for the ASVspoof-2019 LA, ASVspoof-2021 LA, and Arabic spoofing datasets. The comparison is represented in Table 8. RawNet2 processed raw audio signals, whereas other baseline models employed conventional feature-based approaches. It can be observed from Table 8 that the proposed method achieved the lowest min-tDCF and EER for all datasets (ASVspoof-2019, ASVspoof-2021 LA, and Arabic spoofing datasets), compared to the baseline approaches. For ASVspoof-2019 and 2021, the min-tDCF is improved by 0.01 and 0.2, respectively, compared to the best-performing LFCC-LCNN baseline. Moreover, for the Arabic spoofing dataset, the proposed PDK-Net outperformed the other baseline models and improved the EER by 0.9, compared to the best-performing baseline LFCC-LCNN. Hence, the proposed method has better overall performance and improved generalization compared to the baseline methods for detecting logical access attacks. Additionally, the model has enhanced performance for the detection of Arabic spoofing attacks. The outcomes of this experiment reveal the significance of our method for spoofing detection in English and Arabic languages over the ASVspoof baseline countermeasures.

**Table 8:** Comparison with baseline models for ASVspoof and Arabic spoofing datasets.

Models	ASVspoof-2019 LA		ASVspoof-2021 LA		Arabic Spoofing Dataset
	min_tDCF	EER (%)	min_tDCF	EER (%)	EER (%)
Baseline—LFCC-GMM	0.212	8.09	0.5758	19.30	16.98
Baseline—CQCC-GMM	0.237	9.57	0.4974	15.62	18.23
Baseline—LFCC-LCNN	0.100	5.06	0.3445	9.26	8.96
Baseline—RawNet2	0.129	4.66	0.4257	9.50	9.75
PDK-Net (proposed)	0.09	4.55	0.144	6.113	8.06

#### Comparison with existing approaches

To analyze the effectiveness of our method against the existing approaches, a comparison is performed with^16,17,21,29,31,36,37,41,62–66^ utilizing ASVspoof-2019 and 2021 LA datasets. Specifically, a comprehensive set of methods, including conventional approaches, CNN-based methods, self-supervised, and transformer-based framework are included in the comparative evaluation. Table 9 represents the performance comparison in terms of min-tDCF and EER. The comparison reveals the comparable performance of our method on the ASVspoof-2019 LA dataset. Our method performs better than the existing approaches^36,37,41,64,66^ with the min-tDCF of 0.09. However, the approach^65^ is the best performer with a min-tDCF of 0.011 that fused the features from HuBERT and WavLM, classifying through a NeXt-Time Delay Neural Network (NeXt-TDNN). For ASVspoof-2021 LA, our model outperforms the comparative approaches with a min-tDCF of 0.14. This highlights the improved generalizability of the proposed PDK-Net for LA attacks, compared to the existing approaches. Moreover, for the detailed comparative analysis, the PDK-Net performance for VC, TTS, and algorithm-wise attacks (ASVspoof-2019 LA) is also compared in the following subsections.

**Table 9:** Comparison with existing methods for the ASVspoof LA datasets.

Methods	ASVspoof-2019 LA		ASVspoof-2021 LA	
	min-tDCF	EER (%)	min-tDCF	EER (%)
RawNet2-based meta-learning framework^16^	0.028	0.87	–	–
VGGish^21^	0.030	0.07	–	–
Physiological-physical feature fusion-based approach^31^	0.074	2.82	–	–
Global modulation features-based model^36^	0.139	6.32	–	–
CNN-Transformer-based framework^37^	0.156	7.52	–	–
OpenSmile—DNN^64^	0.21	6.67	–	–
OpenSmile—SVM^64^	0.22	7.71	–	–
SpotNet^17^	0.045	–	–	–
Cochleagram with ViT^66^	0.11	6.94	–	–
WavLM—Ecapa^63^	0.022	0.80	0.372	6.68
HuBERT—Ecapa^63^	0.031	1.05	0.539	12.55
DeepLASD^41^	0.122	5.27	0.4250	12.76
HuBERT—WavLM with NeXt-TDNN^65^	0.011	–	–	6.56
Wav2Vec2—AASIST^62^	–	–	0.217	1.19
Audio-text-emotion correlation-based multi-view features^29^	–	–	0.241	2.00
PDK-Net (proposed)	0.09	4.55	0.144	6.11

##### Comparison of VC and TTS attacks

The purpose of this comparative study is to investigate the proposed method’s performance for the detection of VC and TTS attacks against the existing approaches. Table 10 reports the comparison with approaches^21,41,67,68^ for VC and TTS attack detection. It is observed that the proposed PDK-Net is the second-best performer with a min-tDCF of 0.21 and 0.023 for VC and TTS attacks, respectively. For the TTS attacks, the top performer is^67^, attaining the lowest min-tDCF of 0.016; however, method^41^ is the best performer for VC attacks, with a min-tDCF of 0.15. This analysis reveals the effectiveness of the proposed PDK-Net for the detection of TTS and challenging VC attacks, compared to the existing approaches^21,41,67,68^.

**Table 10:** Comparative analysis for VC and TTS attacks (ASVspoof-2019 LA).

Methods	VC Attacks		TTS Attacks	
	min-tDCF	EER (%)	min-tDCF	EER (%)
VGGish^21^	0.40	1.30	0.040	0.04
CLS-LBP^67^	0.41	20.31	0.016	0.64
RawNet2^68^	0.66	18.34	0.068	0.55
DeepLASD^41^	0.15	6.27	0.144	6.69
PDK-Net (Proposed)	0.21	8.80	0.023	0.80

##### Comparison for algorithm-wise attacks

We also conducted a comparative analysis of algorithm-wise attacks to show the PDK-Net performance for each attack against the existing approaches. The comparative analysis for algorithm-wise attacks is shown in Table 11, and the best results are highlighted. We utilized the primary metric min-tDCF to compare the performance with the methods^21,28,68^. The proposed PDK-Net method attained the best min-tDCF for A09 (TTS), A13, A14 (TTS-VC), A17, A18, and A19 (VC) algorithms. It can be noticed that for all the voice conversion attacks (A17-A18), our approach has attained the lowest min-tDCF. RawNet2 previously claimed one of the best published results (min-tDCF of 0.181) for the challenging A17 attack; *however, it is to be noted that the proposed PDK-Net outperforms RawNet2 with a min-tDCF of 0.176.* Overall, the proposed method outperforms the existing approaches for some of the unknown attacks; however, it is the second-best performer for the rest of the attacks, in terms of min-tDCF (the primary evaluation metric for the spoofing detection task). It is important to mention that our method achieved the best results over comparative methods on the most challenging attacks, A17 and A18. Thus, the comparative study proved the robustness of the PDK-Net for standalone spoofing attack detection.

**Table 11:** Comparative analysis for Algorithm-wise attacks in terms of min-tDCF.

ASV2019—LA	PDK-Net (Proposed)	RawNet2^68^	BTS-E^28^	VGGish^21^
A07	0.026	0.038	0.51	**0.008**
A08	0.058	0.111	2.41	**0.009**
A09	**0.008**	0.035	0.49	0.339
A10	0.039	0.049	0.89	**0.010**
A11	0.022	0.050	0.61	**0.010**
A12	0.031	0.044	0.65	**0.019**
A13	**0.003**	0.046	0.53	0.007
A14	**0.008**	0.038	0.53	0.019
A15	0.029	0.038	0.67	**0.011**
A16	0.024	0.046	1.41	**0.012**
A17	**0.176**	0.181	–	0.592
A18	**0.526**	0.528	–	0.581
A19	**0.041**	0.068	–	0.590

Bold values denote the best performance (lowest min-tCDF).

#### Comparison with challenge participants

The goal of this comparative study is to evaluate the effectiveness of the proposed method against the challenge participants of the ASVspoof-2021 challenge. For this, the performance of the model PDK-Net is compared with all the participants utilizing the ASVspoof-2021 LA and DF datasets. We used min-tDCF for comparison of the ASVspoof-2021 LA dataset, whereas for the ASVspoof-2021 DF dataset, EER is utilized. The comparative results against the top 5 challenge submissions are shown in Table 12.

**Table 12:** Comparison with challenge participants.

ASVspoof-2021 LA dataset		ASVspoof-2021 DF dataset	
Methods	min-tDCF	Methods	EER (%)
T23	0.218	T23	15.64
T35	0.248	T20	16.05
T19	0.249	T08	18.30
T36	0.253	T06	19.01
T20	0.261	T22	19.22
PDK-Net (Proposed)	0.144	PDK-Net (Proposed)	17.55

From Table 12, it can be observed that T23 is the best performer among challenge participants on ASVspoof-2021 LA and DF datasets, with min-tDCF and EER of 0.218 and 15.64, respectively. However, it is important to highlight that the proposed method attained the best min-tDCF of 0.144 on the ASVspoof-2021 LA dataset, among all 41 challenge participants. Also, our PDK-Net is the third-best performer with an EER of 17.55, amongst 33 challenge submissions for the ASVspoof-2021 DF dataset. This comparative study reveals the effectiveness of the proposed method for the detection of unseen logical access and deepfake attacks.

## Discussion

In this generative AI era, audio deepfakes pose a significant challenge due to their potential usage for fraud, disinformation, and other malicious activities. Along with the evolving deepfake generation technology, efforts have been made to contribute to improving detection algorithms to mitigate the negative impact of this emerging threat. The literature demonstrates the existence of a research gap regarding the lack of Arabic spoofing detection, enhancement of generalization aptitude, effectiveness of the spoofing detection models against audio perturbations, and the performance for partially spoofed audio samples. Additionally, the need for performance improvement is highlighted for unknown spoofing attacks, such as sophisticated deepfake attacks. In this paper, the presented PDK-Net framework is one of the efforts towards the reliable detection of spoofed audio samples, particularly in the scenario of unknown attacks and Arabic spoofed samples. The proposed method utilizes the approach of supplementing the multi-view acoustic–phonetic features with deep features. The main goal is to learn and extract comprehensive and generalized feature representations that enhance the PDK-Net’s aptitude for the detection of unknown spoofing attacks. Along with that, we also incorporated the cutting-edge KAN classifier, leveraging the proposed framework with the learning of adaptive univariate functions over feature dimensions, hence aiding in the performance improvement across different corpora.

The reliability and effectiveness of our PDK-Net are validated through rigorous experiments performed utilizing our own Arabic spoofing dataset and the standard datasets (ASVspoof-2019 LA, ASVspoof-2021 LA and DF, and partial spoof datasets). Along with the performance enhancement against the baseline methods for overall ASVspoof-2019 and 2021 LA attacks, it also attained improved performance for detecting spoofed Arabic audio samples (Table8). Moreover, the PDK-Net also attained notable results for the detection of TTS and VC attacks (Table 10, 11). This highlights the ability of the proposed approach to accurately identify the dynamic attributes of synthetic voices, lacking the natural acoustic variations that exist in human speech. In the case of algorithm-wise evaluation, it is to be noted that our PDK-Net attains one of the best results with a min-tDCF of 0.176 for detecting the most challenging A17 attack. A17 is also considered the worst case for baselines and ASVspoof-2019 challenge submissions. Furthermore, it is also important to highlight that the proposed PDK-Net achieved the best min-tDCF of 0.144, among the comparative methods (Table 9) and all the 41 participants for LA attacks in the ASVspoof-2021 challenge (Table 12). This demonstrates the aptitude of the proposed model for the detection of unseen spoofing attacks with varied transmission artifacts and codec compressions. The proposed PDK-Net also attained the third-best EER of 17.55, among all the 33 challenge submissions for ASVspoof-2021 DF attacks (Table 12). However, the detection performance for DF attacks remains suboptimal, which can be improved in future research. The cross-corpora performance of PDK-Net for the partially spoofed audio samples is also satisfactory. These results indicate the ability of the proposed framework for the known and unknown spoofing attacks in the scenario of cross-corpora evaluation. Thus, highlighting the great generalization aptitude of the PDK-Net, mainly due to the generalized and deep multi-view feature learning from multiple dimensions, including deep features, phoneme duration, and wave2vec dimension. Performance on perturbed audio samples (Table 4) using the ASVspoof-2019 LA dataset signifies the impact of small audio perturbations on the model’s effectiveness. In future research, the model’s robustness against audio perturbations can be improved by implementing strategies like data augmentation techniques, preprocessing, and denoising input and adversarial training, where the model is exposed to the perturbed samples during training.

The improved performance and notable generalization aptitude ensure that the proposed framework can effectively capture the dynamic speech attributes and subtle variations among spoofed and bonafide utterances. Furthermore, the results, particularly on the ASVspoof-2021 LA dataset, highlight the PDK-Net’s ability to handle various encoding and transmission artifacts, channel variations, and diverse codec compressions. However, the performance needs to be further improved for the detection of the deepfake utterances of the ASVspoof-2021 DF dataset. While the proposed framework demonstrates notable performance, its computational complexity is relatively high, resulting in longer training and inference times. Future research directions involve optimizing the framework to reduce computational complexity and performance enhancement for ASVspoof-2021 DF dataset, adversarial attacks, and challenging A17 and A18 logical access attacks. Another future direction is the development of a dataset with adversarial attacks on utterances, to provide a benchmark for assessing the approaches on adversarial attacked audio samples.

## Conclusion

This paper has presented a novel dual-channel framework, Phonetic-DeepKANet, incorporating deep and multi-view acoustic–phonetic features with a KAN classifier, to combat voice spoofing threats. A novel Arabic audio spoofing dataset is also introduced to accelerate research regarding the detection of spoofing attacks in the Arabic language. For our PDK-Net’s assessment, rigorous experiments were performed utilizing our Arabic spoofing dataset, ASVspoof-2019 LA, ASVspoof-2021 LA and DF, and partial spoof datasets. The cross-corpora results validate the effectiveness and generalizability of our method for the detection of partial spoofing, voice conversion, TTS synthesis, and deepfakes audios involving compression attacks. Specifically, our PDK-Net performed the best on the ASVspoof-2021 LA and third best on the ASVspoof-2021 DF datasets, among all the participating methods in the ASVspoof-2021 challenge. Also, it demonstrates effective detection for the most challenging unknown VC attacks (A17 and A18) and satisfactory performance for perturbed audio samples. However, the detection performance for deepfake attacks and perturbed audio samples needs further improvement.

## Data Availability

Data is publicly available and cited in the paper.
